# Molecular guidelines for promising antimicrobial agents

**DOI:** 10.1038/s41598-024-55418-6

**Published:** 2024-02-26

**Authors:** Mateusz Rzycki, Marta Gładysiewicz-Kudrawiec, Sebastian Kraszewski

**Affiliations:** 1 Department of Biomedical Engineering, Wroclaw University of Science and Technology, 50-370 Wroclaw, Poland; 2 Department of Experimental Physics, Wroclaw University of Science and Technology, 50-370 Wroclaw, Poland

**Keywords:** Machine learning, Virtual drug screening, Computational biology and bioinformatics, Functional clustering, Machine learning, Virtual drug screening, Computational biology and bioinformatics, Functional clustering

## Abstract

Antimicrobial resistance presents a pressing challenge to public health, which requires the search for novel antimicrobial agents. Various experimental and theoretical methods are employed to understand drug-target interactions and propose multistep solutions. Nonetheless, efficient screening of drug databases requires rapid and precise numerical analysis to validate antimicrobial efficacy. *Diptool* addresses this need by predicting free energy barriers and local minima for drug translocation across lipid membranes. In the current study employing *Diptool* free energy predictions, the thermodynamic commonalities between selected antimicrobial molecules were characterized and investigated. To this end, various clustering methods were used to identify promising groups with antimicrobial activity. Furthermore, the molecular fingerprinting and machine learning approach (ML) revealed common structural elements and physicochemical parameters in these clusters, such as long carbon chains, charged ammonium groups, and low dipole moments. This led to the establishment of guidelines for the selection of effective antimicrobial candidates based on partition coefficients (logP) and molecular mass ranges. These guidelines were implemented within the Reinforcement Learning for Structural Evolution (*ReLeaSE*) framework, generating new chemicals with desired properties. Interestingly, * ReLeaSE* produced molecules with structural profiles similar to the antimicrobial agents tested, confirming the importance of the identified features. In conclusion, this study demonstrates the ability of molecular fingerprinting and AI-driven methods to identify promising antimicrobial agents with a broad range of properties. These findings deliver substantial implications for the development of antimicrobial drugs and the ongoing battle against antibiotic-resistant bacteria.

## Introduction

Antimicrobial resistance stands as one of the most significant challenges facing the medical and scientific communities^1^. As emphasized by the World Health Organization (WHO), antimicrobial resistance poses an urgent threat to public health, with the potential to render previously effective antibiotics powerless^2^. To address this growing issue, researchers are actively pursuing the development of novel antibiotics and antimicrobial agents. This pursuit extends beyond mere discovery events. It requires extensive evaluation and screening procedures for precise characterization of molecule with the definition of potential targets.

Understanding the passage of these agents through biological membranes is essential, as it directly influences their pharmacokinetics and pharmacodynamics^3^. To ensure a comprehensive understanding, the evaluations of the newly discovered agents encompass a range of methods, including *in silico, in vitro* or *in vivo* approaches.^4^. Rapid numerical analyses, which include characterizing compound interactions with potential targets, serve as critical support for the experimental validation and optimization of the molecular conformation. It is important to note that these agents utilize different mechanisms of action, including the modulation of membranes that correspond to the carpet model, barrel stave, and pore formation^5^.

Numerous techniques are available to estimate the level of interaction between a compound and a lipid membrane, including molecular dynamics simulations^6,7^, Langmuir-Blodgett trough^8–10^, differential scanning calorimetry^11^, fluorescence spectroscopy^12^ as well as traditional tests such as Minimum Inhibitory Concentration (MIC)^13^ or Zone of Inhibition^14^. These methods offer valuable insights into the interaction between compounds and membranes, a fundamental aspect of the pharmacokinetics of newly developed antimicrobial agents.

However, assessing the antimicrobial potential of a compound is not always straightforward. This underscores the growing need for software tools that can quickly and accurately predict their activity. Existing theoretical methods, such as molecular dynamics (MD) and molecular mechanics/Poisson−Boltzmann surface area (MM/PBSA)^15^ approaches, prove to be well-suited for this task^16^. MD simulations can provide detailed information on the dynamics and physical properties of molecular systems, such as drug-membrane interactions^17^. However, these can be computationally demanding and time consuming. Therefore, its application in high-throughput screening contexts and available timescales is limited^18^. On the other hand, the MM/PBSA method provides an accelerated estimation of binding free energies, which is a critical aspect in drug design. Although MM/PBSA is less computationally demanding than MD, it may offer less detailed information and sometimes oversimplify complex molecular interactions^19,20^. Therefore, they are not always suitable for the rapid screening of numerous compounds. The development of efficient software tools is essential to identify potential candidates for further exploration while simultaneously limiting the time and cost associated with drug discovery^21^. *Diptool*^22^ is specialized software that addresses this knowledge gap. It quickly assesses the interaction energy between a compound and a bacterial target, facilitating the rapid identification of the new antimicrobial agents’ pharmacokinetics. The computational core of *Diptool* is rooted in substantial interactions derived from comprehensive Structure-Activity Relationship (SAR) studies against Gram-negative and Gram-positive bacteria^22^. This approach enables *Diptool* to assess particle activity based on their ability to overcome thermodynamic barriers imposed by bacterial membranes.

In this study, we explored the thermodynamic commonalities between 70 antimicrobial molecules from diverse families, despite their limited structural resemblance. To achieve this goal, we harnessed *Diptool* free energy and clustering techniques to effectively group agents and focus our analysis towards clusters exhibiting promising antimicrobial activity. The improved * Diptool* algorithm offers the ability to generate more realistic free energy profiles, resulting in a more precise depiction of particle behavior within the lipid environment.

Subsequently, through a comprehensive analysis of similarities using physicochemical fingerprints^23^, we identified shared structural elements and parameters within the clusters. Our findings suggest that molecules exhibiting potential antimicrobial activity often possess long carbon-chain backbones^24^, charged ammonium groups^25^, and low dipole moments. Furthermore, considering the entirety of selected molecules, their masses and logP values typically fall within the range of 490-610 g/mol and 8-11, respectively.

Armed with these insights, we implemented the derived guidelines into Reinforcement Learning for Structural Evolution (*ReLeaSE*)^26^, an Artificial Intelligence (AI) framework comprising two deep neural networks, generative and predictive. These networks were specifically trained to generate novel targeted chemicals with desired properties. Interestingly, *ReLeaSE* successfully generated molecules with structural profiles similar to the originally tested antimicrobial agents. This underscores the significance of the identified structural and physicochemical features.

The combination of advanced computational techniques, molecular fingerprinting and AI-driven approaches demonstrated in this study offers bright guidelines for discovering potent antimicrobial agents with diverse and promising properties.

## Results and discussion

### Diptool free energy

In this paper, we employed the updated *Diptool* software that can more accurately reflect the free energy barriers and local minima. *Diptool* was initially developed to screen large databases to accelerate the process of detecting alternative and promising antimicrobial agents^22^. To this end, we made an attempt to characterize selected antimicrobial molecules considering their thermodynamic properties. We also aimed to propose their classification on the basis of the interaction energy with membranes, not only structural properties. Such energy predictions can indicate the antimicrobial potential of the compounds and suggest the ability to combat resistant bacteria.

**Figure 1:**
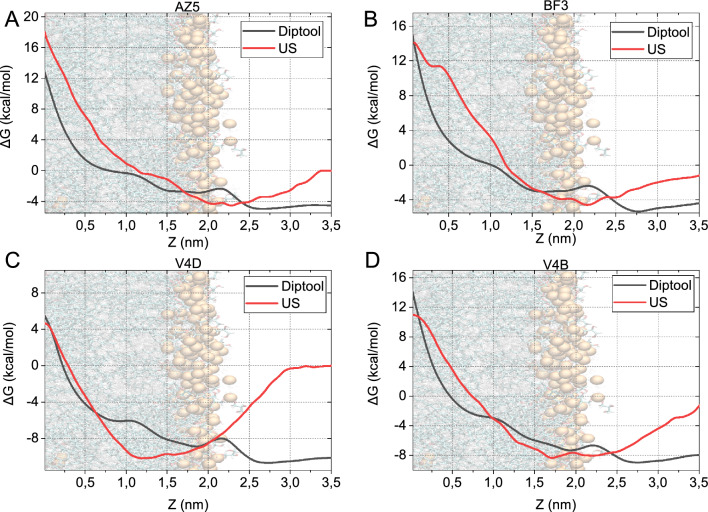
Diptool free energy plots compared with umbrella sampling from molecular dynamics. The figure showcases four representative compounds: (**A**) AZ5, (**B**) BF3, (**C**) V4D, (**D**) V4B. An illustrative location of the membrane is highlighted on each panel with the appropriate marking of headgroups (orange phosphorus atoms) and tails (shades of grey) regions.

First, we characterized the behavior of the agent on the membranes with * Diptool* energy predictions. Here, we extracted all of the molecules’ dipole moments based on their structures in low-energy states. This optimization allowed to simplify and accelerate the whole *Diptool* procedure and input generation, while still ensuring the appropriate agent’s behavior. The *Diptool* free energy plots were compared with the umbrella sampling (US) method from molecular dynamics (see Fig. 1). Four representative compounds with high energies were selected to present the *Diptool* prediction accuracy. AZ5 is a molecule with incorporated N-CH3 substituents (AZA) in the structure^27^, BF3 is bifunctional cationic surfactant^28^, V4D and V4B are bis(alkylammonium) dichlorides, from the family of Quaternary Ammonium Salts (QAS)^29^. The free energy plots can be recognized as a common type according to the classification proposed by Neale  & Pomes based on small molecules’ interactions with membranes^30^. All molecules encounter high-energy barriers when approaching the membrane center, hence the energy at the bilayer midpoint exceeds that observed in bulk water. It may suggest that the molecules preferentially settle on the bilayer surface or can spontaneously enter the inner membrane and anchor in the lipid headgroup regions. The latter can be observed with the V4D and V4B agents (Fig. 1C,D) reaching the global minimum at -10 kcal/mol and -8 kcal/mol from the water baseline, respectively. AZ5 and BF3 potential of mean force (PMF) (Fig. 1A,B) indicated reduced affinity toward the membrane center compared to V4D and V4B. The location of their global minimum $\sim$-4 kcal/mol lies in the hydrophilic headgroup region. Thus, we predict that AZ5 and BF3 can bind tightly to the polar bilayer surface. Their further movement toward the membrane center is not favorable and results in high energy barriers reaching 22 kcal/mol and 18 kcal/mol, respectively. Regarding the V4D and V4B agents, the thermodynamic ’sweet spots’ are located below the headgroups in the carbonyl region at 1.6nm. Onward movement along the bilayer core resulted in overcoming the high energy barriers at 17 kcal/mol and 19 kcal/mol, respectively. We predict that the minor membrane ondulations observed with BF3 and V4B near the membrane center are due to the local conformation of the molecule caused by the transition across the energy barrier.

In the dipole system representation, the *Diptool* free energy maintains a trend similar to that observed using US in all-atom MD. Membrane barriers have been well reproduced; however, in most cases local minima are shifted on average 5Å. This offset is related to short-range electrostatic interactions between dipole headgroups and agents. An additional negative charge held in the polar lipids in conjunction with the cationic character of the tested agents, results in a slight energy increase at the headgroup level. In addition, this arises from large differences in the interacting agent and the membrane dipoles (z-vector component) leading to shifts in the energy diagram.

*Diptool* recognized AZ5 and BF3 global minimum at $\sim$-5 kcal/mol and the total free energy barrier at $\sim$18 kcal/mol and $\sim$19 kcal/mol in the membrane center, respectively. Considering the location of the local minimum, the molecules should bind close to the membrane surface, and further movement toward the center of the membrane is energetically unfavorable. Additionally, slight structure differences between V4D and V4B agents remain visible in the energy plots. V4B is partially larger with greater mass and thus may generate a higher energy barrier. PMFs from the US suggest that both V4B and V4D preferentially anchor below the headgroup region, in the carbonyl-glycerol region. It should be noted that the behavior of the molecules in the bulk water may vary between *Diptool* and US method, as the water interaction sites are omitted. We adopt the implicit solvent representation considering the permittivity and viscosity of the medium. That results in underestimations in the extracellular space. Therefore, we focus on membrane surface events, local minima, and total energy barriers.

### Clustering methods

**Figure 2:**
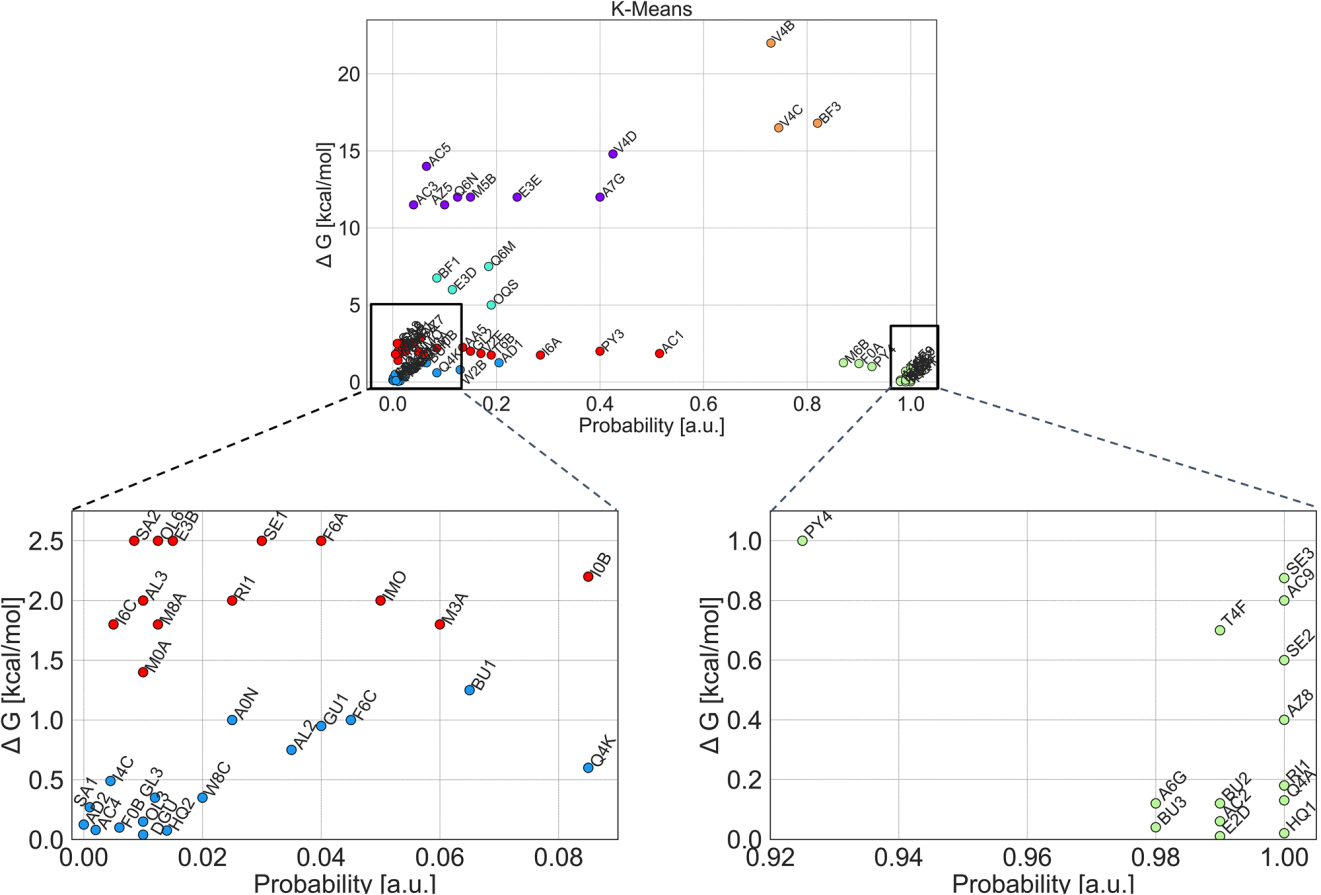
K-means clustering of all antimicrobial candidates collected in the study based on their Diptool dG and probability of entry. The clustering process identified six distinct groups, sequentially marked with colors: orange, green, violet, teal, red, and blue. The two most promising groups: the first (orange) and the second (green) have been selected for the subsequent investigation and thoroughly analyzed.

Considering the limited literature discussion on specific structural elements affecting the antimicrobial activity of the tested molecules, we decided to identify and cluster agents with thermodynamic similarities and characterize them. To this end, we employed the *Diptool* results and Python *scikit-learn* package^31^. For efficient and consistent data analysis only dipole moments (X,Y,Z and total), *Diptool* dG, *Diptool* probability, SMILES, mass, and logP classifiers were taken into account. Here, *Diptool* dG is interpreted as the absolute difference between the molecule in the water (a) and lipid (b) phase $(dG=G_b-G_a)$. *Diptool* probability determines the potential for proper binding to the membrane core. It is reported as the ratio of runs with correctly anchored agents in the membrane to all simulations performed. Thus, the results from *Diptool* enable the evaluation of the molecule’s binding capacity to the membrane based on the given probability function. The k-means (see Fig. 2) and other algorithms (agglomerative and spectral clustering, affinity propagation, see Figs. S1, S2, S3) were used to combine *Diptool* probability and *Diptool* free energy. Given the selection, 6-8 clusters were identified, respectively.

Despite the various number of clusters, many compounds have been grouped in a similar manner among algorithms. The greatest amount of shuffling in groups is visible in the regions of points with the highest density (marked red and blue in Fig. 2). Primarily, it concerns agents with low free energy and probability of entry. Differences may arise from the assumptions of the algorithms, including the tendency of k-means to produce clusters of equal size. However, considering the promising antimicrobial properties, agents with a high binding coefficient (high probability) and a low-energy barrier are the most desirable. The compound should pose a high probability of entry, yet a low transition-free energy. On the other hand, molecules with high free energy are equally valuable as they can bind tightly to the membrane headgroups, leading to pore formation and membrane disruption. Thus, the most interesting clusters lay at the edge of the probability axis, posing a high tendency to bind (green and orange in Fig. 2). The agents collected within the remaining clusters are not characterized by a high probability of entry. Therefore, in our study, all clusters with an entry-level probability below 0.6 were excluded. The combination of *Diptool* predictions and clustering methods allowed us to identify 19 highly potent candidates. These compounds were further analyzed to verify their common features and identify potentially promising properties. The first cluster included molecules with low entry energy, while the second one - with high. Detailed information on the selected candidates is listed in the attached .xlsx file in the Supporting Information.

### Analysis of the similarities between the selected candidates

To identify promising agents and their features, we employed several clustering models to group selected molecules based on their *Diptool* free energies. To this end, we employ the * i.a.* k-means method and select two groups of agents whose behavior heralds promising features for potent candidates. In this section, we conducted a comprehensive similarity analysis of the molecular structures within each cluster to explain the close thermodynamic nature of the agents. To perform this analysis, we used the *RDkit* library, which offers a wide range of descriptors to capture diverse aspects of molecular properties. By comparing the molecular structures and their corresponding descriptors, we aimed to identify common features and patterns that could potentially contribute to their antimicrobial properties.

In the first cluster (orange - see Fig. 2), 3 candidates (BF3, V4C, V4B) were collected that exhibited elevated *Diptool* free energy with a high probability of entry. However, the membrane energy barrier is too high for them to spontaneously reach the bilayer hydrophobic core. These bind at the lipid headgroups level, thus can induce membrane defects acting similarly to some antimicrobial peptides^32^. All three molecules share a fundamental structural framework comprising an alkyl chain backbone composed of a linear arrangement of carbon atoms. Hydrophobic carbon chains range from 14 to 16 carbons in length. Additionally, they possess a common characteristic motif consisting of two quaternary amine groups separated by a spacer segment. BF3, V4B, and V4C have a double bond within their carbon chains, although in different arrangements. The latter agents share a similar structure with shorter alkyl chains between the amine groups, but generally lack the carbonyl moieties observed in BF3. Despite numerous structural similarities and differences, molecules have been classified within the same cluster based on their high probabilities of entry and high *Diptool* free energy. It is worth noting that all molecules also exhibit high dipole moment values compared to the others, which could influence the system’s thermodynamics. Dipole moments are one of the few experimental parameters that directly correlate with the charge distribution in the molecule^33^. Moreover, it is suggested that many antimicrobial agents exhibit a strong relationship between activity and the dipole moment^34,35^. Noteworthy, most of the studies examining the relationship between dipole moment and the biological activity of compounds have predominantly indicated good antimicrobial efficacy exhibited at lower dipole moments^33,34,36^. Consequently, molecules collected within cluster 1 are more likely to act as mediators or linkers, facilitating the access and anchoring of agents with higher antimicrobial activity to the membrane.

In the second cluster of agents (green - see Fig. 2), 16 candidates (A6G AC2 BU3 HQ1 AC9 PY4 T4F SE3 RI1 Q4A M6B F0A E2D AZ8 BU2 SE2) were collected that exhibited low *Diptool* free energy with a high probability of entry. This set of features is particularly desirable, providing an opportunity for spontaneous particle embedding in the bilayer interior. This suggests that upon surpassing a small energy barrier, further penetration of the agent toward the bilayer interior may follow with minimal resistance of free energy^37^. The mode of action of the agents these can focus on emulsification of membrane components or damaging the ion channels, resulting in leakage of the cytoplasm and cell death^38–40^. In addition, notable structural similarities within a group may remain essential for understanding agent properties and potential applications. All molecules in the cluster possess an extended hydrophobic carbon chain backbone (12–16 carbon atoms) providing the amphiphilic properties of the molecules. An increased chain length can enhance the interaction between the active molecule and the bacterial cell membrane, leading to improved antibacterial activity^41,42^. The presence of positively charged quaternary ammonium groups (N$^+$) is another common feature among agents in the cluster. The positive charge facilitates electrostatic interactions with negatively charged components on the bacterial cell surface, disrupting the cell membrane and exerting bactericidal effects^40,43^. Based on our findings, we did not observe a notable correlation between the reduction in net charge and the restricted thermodynamic activity, as discussed in relation to antibacterial activity by Jiang et al.^44^. Another common feature of a couple of clustered molecules is the presence of aromatic rings or nitrogen-containing heterocycles, particularly benzene and pyridine moieties. These aromatic moieties can participate in $\pi -\pi$ interactions, resulting in effective suppression of bacteria^45,46^. The introduction of specific substituents or functional groups, such as halogen atoms (e.g., fluorine, or chlorine), can modify the lipophilicity or hydrophobicity of the particle structures. These modifications have the potential to enhance the affinity of the agents for bacterial membranes, thus increasing their biological activity. Wu et al.^47^ reported the correlation where the compound with a chlorine atom in between was more active than the other compounds.

It is worth noting that the molecules in the second cluster exhibited reduced dipole moment values compared to those in the first one. Das et al.^33^ found that there exists a significant correlation between the biological activity of numerous compounds and their dipole moment. Our results support this hypothesis, as lower energies (*i.e.* energy barriers), and consequently, higher affinity and potential antimicrobial efficiency are observed at reduced values of the total dipole moment. These findings remain consistent with reports from Asad et al.^48^ where N-propionyl pyrazolines were tested against Gram-positive and negative strains. They found that compounds with reduced dipole moment exhibited increased antimicrobial activity against *S. aureus*, *B. subtilis* and *E. coli* using zone of inhibition assay. Similar conclusions were also drawn from various studies dealing with the antimicrobial activity of sulfonamides^36^, Schiff base structures^49^, $\alpha$-aminophosphonates^50^ or pyrimidine derivatives^51^. The results obtained from extensive investigations utilizing various experimental techniques, including the zone of inhibition and the MIC, demonstrate a strong correlation between increased antibacterial activity and restricted dipole moment. Thus, the molecules collected within the second cluster represent promising candidates for further experimental investigation to validate their effective antimicrobial performance. Furthermore, a low total dipole emerges as a novel selection criterion for antimicrobial agents.

**Figure 3:**
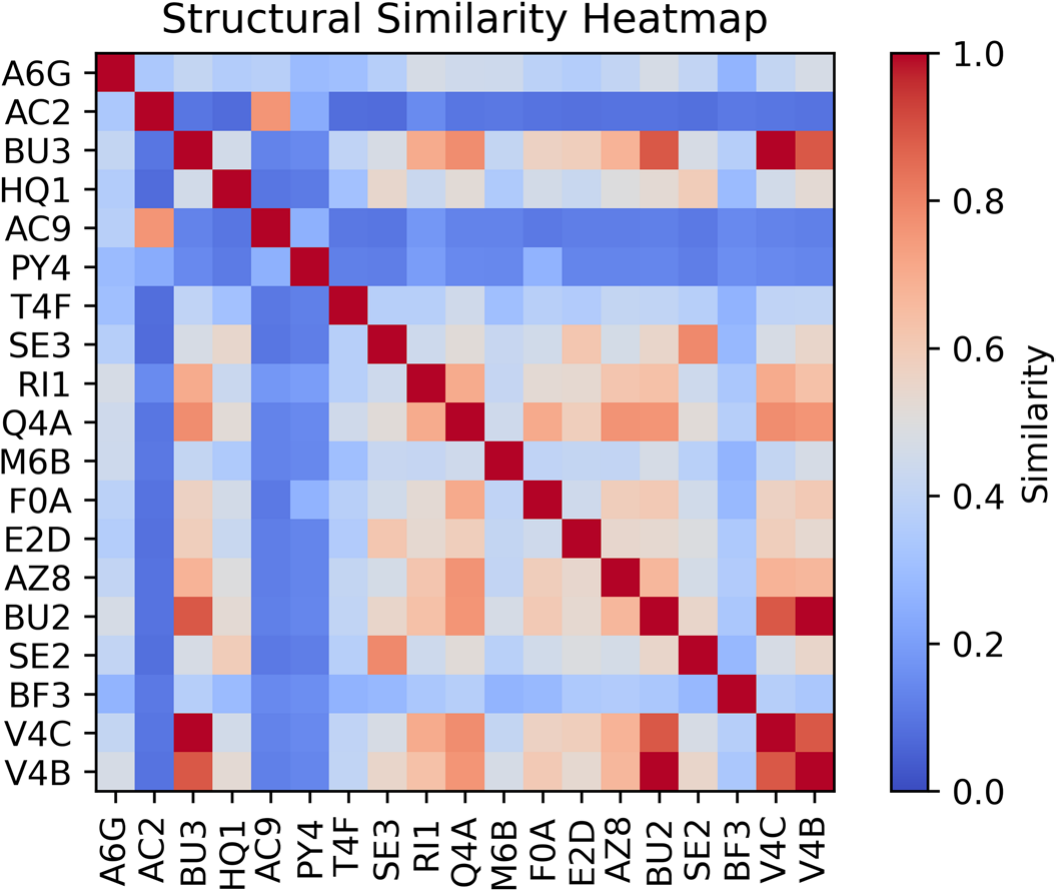
Structural similarity heatmap of selected agents derived from their molecular fingerprints. The heatmap employs a color scale ranging from 0 to 1 to represent the extent of similarity, with a value of 0 and 1 indicating no similarity or complete structural similarity, respectively.

In addition, to facilitate a more comprehensive analysis, we consolidated all of the molecules classified within both clusters into a heatmap of structural similarity. The collected molecules demonstrated striking similarities in their thermodynamic characteristics, despite originating from other structural groups, and bearing distinct functional substituents. To provide a more detailed and complex overview, we used molecular fingerprints to assess the extent of structural similarities among the molecules. To accomplish this, the 3D structures were converted to Morgan fingerprints. These encode atom groups in a binary vector that represents the presence or absence of specific molecular features or substructures^52^. Subsequently, utilizing the generated fingerprints, the Tanimoto matrix was constructed (Fig. 3). The Tanimoto coefficient is a measure of similarity that quantifies the overlap between two fingerprint vectors ranging from 0 to 1 indicating no or complete similarity, respectively.

The lowest structural similarity is exhibited by the AC2, AC9, and PY4 molecules, since the similarity coefficient does not exceed 0.2 on average. The first two agents are derivatives of structurally related groups containing pyridine moieties, displaying a higher pairwise similarity of 0.76. They are distinguished from other agents by their distinctive cyclic construction. PY4 due to its complex conformation with multiple fluorine moieties, also shares low similarities with other agents. On the other hand, the greatest similarities were observed in BU3, Q4A, BU2, V4C, and V4B, since the similarity index indicates above 0.5 on average. As previously, many similarities are observed for analogous molecules such as BU2 and BU3, as well as V4C and V4B. These molecules possess QAS-specific elements in their structure, *i.e.* positively charged nitrogens and long alkyl chains that aim to penetrate the cell membrane and double bond in the spacer. These molecular guidelines are known and have already been proposed for antimicrobial agents, as stated above. Here, the similarities can reach even 0.9 for BU2 and V4C and for BU3 and V4B. The most common features with all collected agents has Q4A. The Tanimoto coefficient exceeded 0.7 for six molecules in the subset. This high similarity arises from the linear nature of the molecules and their shared structure resembling classical quaternary salts.

**Figure 4:**
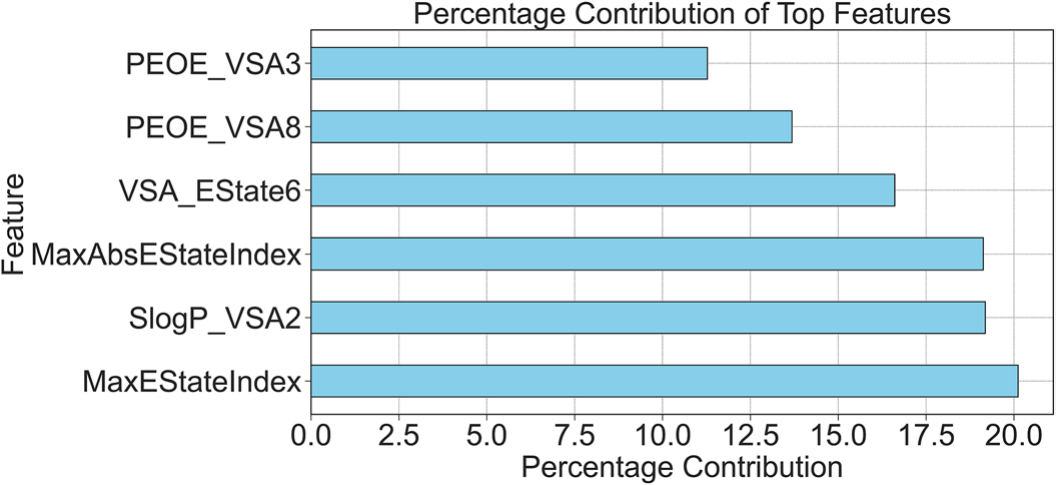
A set of six highly influential descriptors that significantly contribute to the prediction of the probability of membrane penetration ratio from our ML analysis. Most of the highlighted features are related to the electrostatic or electronic properties of tested agents.

To precisely identify the characteristics of promising agents, we used additional machine learning methodology for the analysis of physicochemical descriptors for the *Diptool* dataset. This analysis revealed a set of six highly influential descriptors that contribute significantly to the prediction of the probability of membrane penetration ratio (see Fig. 4). The descriptors capture various aspects of the molecular structure, electron distribution, and functional groups. The analysis revealed that the set of most significant descriptors, identified by their feature indices, include MaxEStateIndex, SlogP_VSA2, MaxAbsEStateIndex, VSA_Estate6, PEOE_VSA8, and PEOE_VSA3. Ranking scores indicated the relative importance of each descriptor, with higher scores corresponding to greater influence on the prediction task.

Among these descriptors, MaxEStateIndex represents diverse electronic properties within the molecule. While it does not directly relate to molecular size, LogP, or molecular dipole implemented within *Diptool* software, it captures important information regarding the distribution of electron states. On the other hand, SlogP_VSA2 contributes to the lipophilic nature of the molecule, making it relevant to directly implemented LogP, which measures the tendency of the molecule to dissolve in lipids. MaxAbsEStateIndex represents the overall electronic properties of the molecule, and indirectly influences properties related to the molecular dipole aspect, such as polarity or reactivity. VSA_Estate6 captures fragment-specific electronic properties, providing insights into the reactivity and chemical interactions associated with specific fragments within the molecule. Additionally, we found two descriptors related to electrostatic interactions. PEOE_VSA8 and PEOE_VSA3 contribute to the calculation of the potential electrostatic energy between the atoms of the molecule, providing valuable information related to the molecular dipole aspect. To summarize, MaxEStateIndex captures diverse electronic properties, SlogP_VSA2 relates to LogP and lipophilicity, MaxAbsEStateIndex influences overall electronic properties and indirectly relates to the molecular dipole aspect, VSA_Estate6 characterizes fragment-specific electronic properties, and PEOE_VSA8 and PEOE_VSA3 capture electrostatic interactions. These descriptors collectively provide valuable information on various aspects of molecular structure, electron distribution, and functional groups. They enhance our understanding of membrane penetration and assist in designing molecules with desired properties for pharmaceutical or biotechnological applications.

**Figure 5:**
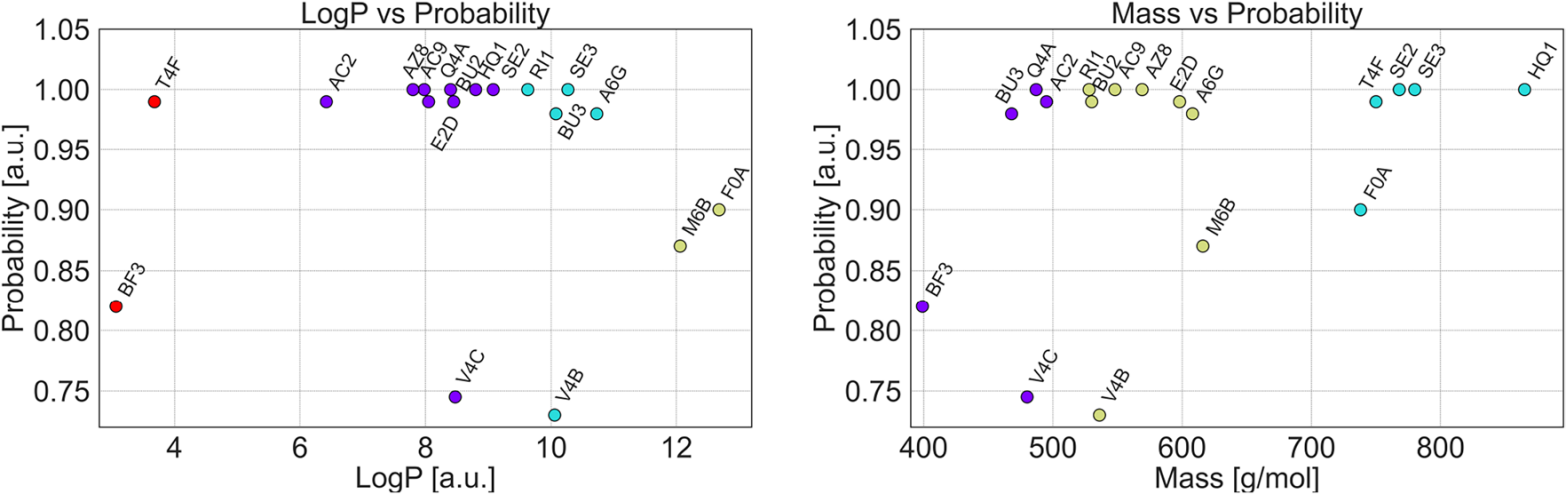
The most promising and selected antimicrobial candidates, identified based on their LogP/mass and Diptool probability of entry. The performed analysis allowed to determine the logP and mass ranges in which prominent molecules were collected. From this analysis, we derived the guidelines for antimicrobial molecules and applied them to the *ReLeaSE* method.

Taking all this into account, we have decided to summarize the selected molecules relative to their logP and mass versus a high probability of entry from *Diptool* (Fig. 5). The relationship between logP and antimicrobial activity has been a subject of interest in medicinal chemistry and drug development^53–55^. It measures the lipophilicity or hydrophobicity of a compound, thus the ability to bind to a membrane. Compounds with lower logP values tend to exhibit a decrease in lipophilicity, which can limit their penetration through lipid-rich bacterial cell membranes^56,57^. Thus, improved membrane permeability can enhance their ability to reach intracellular targets or exert antimicrobial effects. Our studies suggest that most of the agents selected with * Diptool* as good antimicrobial candidates ranged between 8 and 11 in logP. Such values indicate a higher logP compared to OCT or CHX, both recognized for their effective action on membranes^58^. This observation may serve as a refined selection criterion for antimicrobial agents.

Interestingly, the molecular molar mass of a compound can also have an impact on its antimicrobial activity. Although, there is no universal rule or direct correlation between molar mass and antimicrobial activity known in the literature. Nonetheless, larger molecules with higher molar mass *i.e.* analogs with longer alkyl chains, may exhibit reduced antimicrobial activity compared to smaller ones^59^. This could lead to restricted access to intracellular targets or reduced particle penetration, through bending and curling of chains, which in turn hinders electrostatic interactions with bacteria and limits the antibacterial potency^59^. In our subset of agents, the majority of the most active fall between 490-610 g/mol in mass. Surprisingly, it is suggested that there is an optimal size-mass range for antimicrobial compounds. Therefore, particles with the corresponding molar masses display higher activity^60^. Thus, considering the distribution of all agents in the study, the present range contains medium-sized molecules and could be taken as an explicit selection criterion for antimicrobial agents.

**Figure 6:**
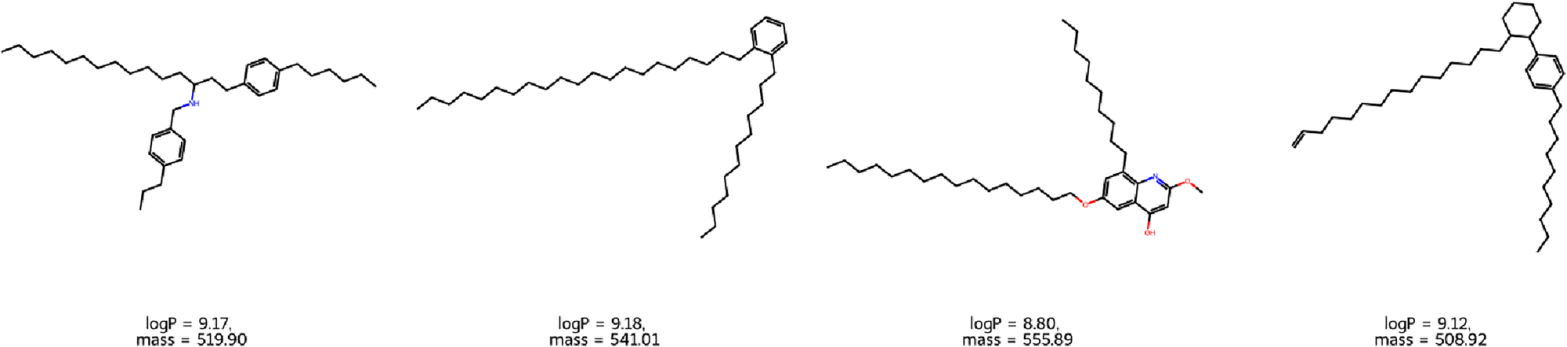
Potential antimicrobial agents generated using the ReLeaSE method, based on the provided guidelines.

Finally, we decided to test our antimicrobial particle guidelines proposed here, based on low total dipole and range-defined logP and molar mass of agents. For this purpose, we implemented the *ReLeaSE*^26^ method to generate novel compounds (using SMILES) of promising candidates. As explained in the *Theory* *and* *Methods* section, the process involved training generative and predictive networks based on the logP dataset provided by the *ReLeaSE* authors. Subsequently, compound creation was selectively biased to match the logP within the range of 8-11. Furthermore, we filter the generated molecules to fall within the molecular mass range of 490-610 g/mol. Finally, the obtained SMILES were converted to 3D structures using *ProNovo* online server (https://novoprolabs.com/tools/smiles2pdb), and then optimized with the PM7 level of theory, using our usual procedure described elsewhere^22^. As a result, we obtained a set of fully AI-formed molecules that exhibit structural similarities to those used in the clustering process. Representative agents with low total dipole moments (<2.1) are shown in Fig. 6, and the entire list is available in Supporting Information Fig. S4.

Notably, newfound agents are equipped with elongated carbon alkyl chains or possess ring substituents, which often enhance antimicrobial activity and were previously discussed. We assume that our new molecular guidelines for antimicrobials proposed here will open up new opportunities for more effective research in the fight against resistant strains.

## Conclusions

In this paper, we utilized *Diptool* to systematically characterize selected antimicrobial compounds based on their thermodynamic properties. This allowed us to propose refined classifications of these agents concerning their interactions with membranes and their potential antimicrobial activity. This approach offers particular value in particle analysis and the development of novel drugs targeting antibiotic-resistant bacterial strains.

First, we analyzed the behavior of the agents on the membranes using * Diptool* energy predictions. *Diptool* free energy plots corresponded well with the umbrella sampling (US) method for all-atom molecular dynamics (MD), confirming the reliability of our approach. The molecules exhibited high-energy barriers when approaching the center of the membrane. This suggests a preference for settling on the surface of the bilayer or, more likely, penetration of the inner membrane and anchoring in the bilayer. We identified several thermodynamic local minima for different drugs, such as V4D and V4B, which showed favorable positioning below the head groups in the carbonyl region. Otherwise, AZ5 and BF3 showed a lower affinity toward the membrane center and likely bound tightly to the polar bilayer surface from the extracellular environment.

Afterward, using clustering methods, we grouped thermodynamically similar compounds to identify potential candidates with promising antimicrobial properties. From a collection of tested clustering approaches, we selected the k-means algorithm for in-depth analysis of the active compounds. The agents were grouped according to *Diptool* membrane penetration probability (internal score) and Gibbs free energy across membrane translocation. This resulted in the identification of 19 highly potent candidates within two clusters. In particular, molecules in the second cluster with high binding coefficients (high probability) and low energy barriers were considered the most desirable as they showed a high tendency to bind to the membrane core. We excluded clusters with a low probability of entry (below 0.6) from the deeper analysis due to the limited level of agent-membrane interaction.

Further analysis focused on two distinct clusters of compounds. Cluster 1 included molecules that exhibited elevated *Diptool* free energy with a high probability of entry, however, the limited ability to spontaneously penetrate the membrane core. These agents showed structural similarities with the alkyl chain backbone, quaternary amine groups, and higher dipole moments. This suggests a potential role as mediators or linkers, facilitating access and anchoring in the bilayer. Cluster 2, on the other hand, included molecules with low *Diptool* free energy and a high probability of entry, suggesting spontaneous embedding of the agents in the bilayer interior. These molecules possessed elongated carbon chains, positively charged quaternary ammonium groups, and aromatic rings or nitrogen-containing heterocycles. These structural elements contributed to both enhanced antimicrobial activity and lower dipole moments. Furthermore, the analysis revealed a significant correlation between reduced dipole moment values and enhanced antimicrobial activity, providing valuable insights for the future development of effective antibacterial agents. This has led us to establish a new molecular criterion for antimicrobial agents.

Structural similarities among the selected candidates were further analyzed using physicochemical fingerprinting, revealing striking similarities despite different structural groups and functional substituents. Machine learning analysis was used to identify six influential molecular descriptors that provide valuable information about the molecular dipole aspect. In particular, they contribute to the prediction of agent activity by providing deep insights into molecular structure, electron distribution, and functional groups. The combination of *Diptool* predictions and clustering methods enabled the establishment of novel and essential molecular guidelines for the selection of promising antimicrobial candidates. First, molecules with logP values between 8 and 11 were identified to have increased lipophilicity, improving their ability to penetrate lipid-rich bacterial cell membranes. Thus, increasing the probability for the agent to reach intracellular targets. Second, the mass of the most active candidates falling in the range of 490-610 g/mol indicates an optimal size range for antimicrobial compounds.

Based on our three novel guidelines for antimicrobial a, we used the Reinforcement Learning for Structural Evolution (*ReLeaSE*) method to generate new compounds (using SMILES) with highlighted logP and masses. The resulting fully AI-formed molecules, sorted by dipole moment, showed structural similarities with the molecules used in the clustering process. They possessed elongated carbon alkyl chains and ring substituents known to enhance antimicrobial activity.

Summarizing our comprehensive study using *Diptool*^22^ has systematically characterized antimicrobial compounds, leading to refined classifications based on their interactions with membranes. We identified thermodynamic ’sweet spots’ for various drugs, indicating potential settling areas on the bilayer surface or within the inner membrane. Our clustering approach successfully pinpointed 19 potent candidates, especially molecules in the second cluster with high binding coefficients and low-energy barriers, which suggest their natural propensity to embed in the bilayer interior. In addition, we established new molecular criteria for antimicrobial agents, notably reduced dipole moments and a specific logP range, which correlate with increased activity. The application of physicochemical fingerprints and machine learning techniques has been instrumental in the identification of six key molecular descriptors that enhance our understanding of agent activity. Furthermore, using the *ReLeaSE* method^26^, we have generated novel compounds, signaling promising avenues for the future development and discovery of effective antibacterial agents. These findings, supported by *Diptool* predictions and AI-driven clustering methods, offer new and valuable guidelines for the development of antimicrobial agents with diverse and promising properties. Our research offers promising implications for the combating of resistant bacteria and for guiding future pharmaceutical or biotechnological applications. Additionally, it enriches the field of drug discovery with innovative methodologies and insights.

## Theory and methods

### Diptool

In this paper, we used the updated version of *Diptool*, a software originally designed to assess the antimicrobial activity of surfactants. Major updates include improvements to the energy function and handling of dipole interactions, the applicable cutoff, and the implementation of the histogram analysis method in the reconstruction of the energy profile. These enhancements now make it a versatile tool for evaluating free energy profiles across various membrane systems. Our primary focus lies in cationic antimicrobial surfactants belonging to the Gemini family, particularly on planar homogenous membranes. Nevertheless, we anticipate successful applications for other molecules, including antimicrobial peptides or chemotherapeutic drugs. The core interactions in *Diptool* rely on electrostatic interactions between a system of dipoles within a medium implicitly described by dielectric permittivity ($\epsilon$) and appropriate viscosity ($\nu$). Each molecule is represented as a sphere with an assigned dipole moment, and the potential energy function considers the dipole-dipole interactions between the lipid moieties and the studied antimicrobial agent. The relevant variables governing these interactions are the dipole moment ($\mu$) and the distances along the z-direction ($z_1$ and $z_2$):

1 $$\begin{aligned} E_{1,2}=- \frac{\mu (z_{1}) \mu (z_{2})}{2 \pi \epsilon _{0}|z_{1}-z_{2}|^{3}}(1-3 \cos ({\theta })) \end{aligned}$$

In our approach, the angle ($\theta$) represents the orientation of the dipole moments. By solving the equations of motion while accounting for the resistance of the medium (viscosity), we assume that the kinetic energy cannot exceed the thermal motion energy (*kT*). However, we encountered the challenge of the exact unknown value of $\theta$ due to molecular simplification as a sphere. Consequently, a slight improvement in the interaction equation was necessary. In our current model, we describe the energy of interaction between the particle and the dipoles in the membrane as a sum:

2 $$\begin{aligned} E_{tot}=-\sum _{i=1}^{N} \frac{\mu (z) \mu (z_{i})}{2 \pi \alpha \epsilon _{0}|z-z_{i}|^{3}} \end{aligned}$$

To address this, we previously introduced the parameter $\alpha$, which empirically corrects the interaction energy based on the relative arrangement of molecules during simulations. The estimated value of $\alpha$ is now derived by considering the extreme positions of the dipoles and calculating the average interaction energy. Here, *N* represents the number of dipoles present in the membrane. The interaction energy obtained through this method is subsequently added to the kinetic one. In addition, we take into account the work done in the given medium, which is related to viscosity. By considering these factors, we determine the Hamiltonian and subsequently the Gibbs free energy as a summation of changes occurring through quasi-equilibrium states $A \rightarrow B$:

3 $$\begin{aligned} \Delta G_{A \rightarrow B} = H_{B}-H_{A} \end{aligned}$$

4 $$\begin{aligned} G(z)= \sum \biggl \langle \frac{\Delta G}{\Delta z} \biggr \rangle \Delta z \end{aligned}$$

The *z* direction represents the reaction coordinate - a *z*-axis projected distance (membrane normal) of the molecule center of mass relative to the membrane center. The simulations are performed under the NPT assumption (constant Number of particles, Pressure, and Temperature). The *Diptool* updated version generates a smoothed energy plot by calculating the average energy values within the specified bins along the *z* coordinate. Firstly, the energy data are processed and normalized to a reference point, which is far from the membrane in bulk water. Subsequently, the mean energy for each bin is calculated using the scipy $binned{\_}statistic$ function^61^. To ensure a smooth representation, a Gaussian smoothing filter is applied to the mean energy values. The resulting plot illustrates the smoothed energy profile along the *z*-coordinate, providing additional valuable insights into the system’s behavior. This approach allows for a visual representation of the overall energy trend, aiding in the analysis and understanding of the system under investigation.

Note that the calculations performed using *Diptool* are limited solely to dipole-dipole interactions. Thus, we do not consider other types of interactions. Another remark concerns solving a second-order differential equation using a modified Verlet algorithm. The accuracy of the method is influenced by the precision of the numerical approximation, in our case, associated with the accuracy of determining the numerical derivative. We employ a three-point scheme with an error of $\delta _{ x, y, z}^2$. In our case, it has been set to 0.01 Å, which seems to yield results with satisfactory precision. The same approach has been applied to the time step, where its value affects the precision of the calculations. However, if the time step is too small, the calculations may take a long time and may not necessarily provide more accurate results. If this step is too large, there is a risk of losing information about significant changes in the considered system. Thus, the simulation time step has been set to $10^{-12}$ s as the optimal range.

### Data preparation

In this paper, we employed the new set of Gemini agents database^6^ and extracted 70 molecules with approx. 10-14 carbon acyl chain length. This selection criterion was adopted since molecules with a given chain length exhibited the most potent antimicrobial and antifungal effect in other studies^62–66^. The selected agents were described with the corresponding aliases, dipole moments, dG of molecule’s transfer across POPG membrane mimicking the bacterial one, membrane penetration probability, mass, and logP (see .xlsx file in Supporting Information).

Dipole moments were determined from the optimized low-energy conformation of the compound using MOPAC software (Molecular Orbital PACkage) with the PM7 method (https://zenodo.org/record/6728590)^67^. To calculate the masses and LogP, SCIGRESS software was used, employing the appropriate three-dimensional coordinates of the molecules [SCIGRESS (2013) Fujitsu Limited, Tokyo, EU 3.3.3]. The simulations were carried out with the * Diptool* package. New releases are available in the GitHub repository: https://github.com/mrzyckiz/Diptool. In the present study, a membrane model consisting of 100% phosphatidylglycerol (POPG - 16:0/18:1) was utilized, as it constitutes a prominent component within bacterial bilayer structures. Membranes of other organisms can also be modeled providing the corresponding dipole moments of the dominant lipids. The area per lipid for PG was set to 63Å$^2$^68^. Given the stochastic nature of *Diptool* simulations, 1000 iterations were performed for each molecule, followed by a histogram analysis approach with a 1Å width step along the membrane normal.

### Clustering methods

In this work, we decided to use several clustering techniques to propose an optimized grouping method founded on agent activity rather than structural fit. So far, molecules have usually been classified due to the presence of the functional group, its derivatives, or a synthesis method. Such classifications do not often provide an optimal way to compare the physicochemical properties of compounds. In our study, we decided to use *Diptool* free-energy output to group structurally different molecules, with similar behavior in the lipid membrane medium. To this end, we used the scikit-learn library^31^ to construct various clustering models, including k-means, affinity propagation, spectral clustering, agglomerative clustering, and DBSCAN, for the selected antimicrobial agents. The k-means method met the best clustering requirements (v-measure, silhouette coefficient) and, hence, was selected for further analysis presented in the main text. However, affinity propagation, spectral, and agglomerative clustering methods also met the threshold requirements of the clustering and therefore were included in the Supporting Materials Figs. S1, S2, and S3 for quality comparison.

### RDkit machine learning library

Molecular descriptors play a crucial role in understanding the physicochemical and biological properties of chemical compounds. They provide quantitative representations of molecular structures, enabling the prediction and classification of various molecular properties. In this study, we employed accessible sets of descriptors to investigate their impact on predicting the properties of the above described *Diptool* dataset. The dataset includes molecules with diverse chemical structures and associated labels reflecting a specific property of dipole moments, dG molecule transfer across membrane, mass, logP, and probability ratio of membrane penetration, which we chose as main predictor for potential membrane decomposition. To perform the analysis, we utilized the *RDKit* 2021.03.2 library in Python for descriptor calculation (https://zenodo.org/record/4750957). The dataset of the molecules tested here was loaded from SMILES, and for all molecules molecular 2048 Morgan fingerprints were extracted for further analysis^69^. Then all 208 available chemical descriptors were calculated for each molecule using the *RDKit* implementation. We then performed random-forest-based feature ranking to identify the most informative descriptors within the dataset. The ranking was based on the feature importance function implemented within the random forest classifier.

### Structural similarity

The identified descriptors cover a wide range of molecular properties and structural characteristics. Here, we consider a dataset with *N* molecular fingerprints, denoted $V_1$, $V_2$, ..., $V_n$. Each fingerprint *V* is a binary vector representing the presence or absence of certain molecular features or substructures. The Tanimoto similarity matrix *M* is an $m{\times }m$ matrix, where each element $x_{ij}$ represents the Tanimoto coefficient between the fingerprint $V_i$ and the fingerprint $V_j$ and is calculated as follows^70^.

5 $$\begin{aligned} T(V_{i},V_{j})= \frac{V_{i} \cap V_{j}}{V_{i}+V_{j}-V_{i} \cap V_{j}} = \frac{N_{c}}{N_{a}+N_{b}-N_{c}} \end{aligned}$$

where *N* represents the number of features (bits set to 1) in objects (*A*, *B*) and *C* is the intersection set. The Tanimoto coefficient ranges from 0 to 1, meaning no or complete similarity between the fingerprints, respectively.

### Reinforcement learning for structural evolution (ReLeaSE)

*ReLeaSE* (Reinforcement Learning for Structural Evolution)^26^ represents a pioneering computational strategy devised for the *de* *novo* design of molecules with predefined properties. This innovative approach leverages the connection of deep learning and reinforcement learning techniques to generate novel chemical structures. The method involves two distinct deep neural networks, generative and predictive models, trained independently, yet seamlessly integrated to facilitate the creation of targeted chemical libraries. Notably, molecules are represented through Simplified Molecular Input Line Entry System (SMILES) strings, ensuring a straightforward and efficient encoding process. Generative models utilize a stack-augmented memory network, enabling the production of chemically viable SMILES strings. Simultaneously, predictive models are developed to estimate the desired properties of the generated compounds, here based mainly on LogP. During the initial phase, the generative and predictive models undergo separate supervised learning, where input-output pairs are provided to train the models effectively. Subsequently, the joint training phase employs reinforcement learning principles to bias the generation of new chemical structures toward those exhibiting the desired physical and/or biological properties. We directly followed the procedure described on GitHub (https://github.com/isayev/ReLeaSE). The *ReLeaSE* method exhibits promise in expedited molecular discovery and targeted compound optimization for various applications, encompassing both single and multiple desired properties. Obtained generated molecules were trimmed by molecular guidelines we derive, getting potential novel agents that may have antibacterial properties.

### Molecular dynamics simulations

The membrane builder in CHARMM-GUI^71^ was used to create a bacterial internal membrane model. The system consisted of 124 PYPE (phosphatidylethanolamine), 24 PYPG (phosphatidylglycerol) and 8 PVCL2 (cardiolipin). The TIP3P water model (40 water molecules per lipid) with 0.22 NaCl solution was utilized reflecting appropriate ions concentration in bacteria. All-atom simulations were performed in GROMACS (v. 2021)^72^ with CHARMM36 force field^73^. The temperature was maintained with the Nose-Hoover thermostat at T=303.15 K and $\tau _t$ = 1 ps time constant^74,75^. Pressure was controlled with a semi-isotropic coupling via Parinello-Rahman barostat at p=1 bar and $\tau _p$= 5 ps^76^. Under the equilibration procedure, the NVT (constant Number of particles, Volume and Temperature) ensemble was applied with 1 fs time step for 250ps. Further, NPT dynamics was applied with the integration step of 2 fs for 5ns. In the equilibration procedure, the positional and dihedral restraints were gradually decreased. The proper production run lasts for 200ns. Van der Waals interactions were cut-off at 1.2nm with a force-based switching function^77^, and long-range electrostatics were evaluated using the particle mesh Ewald (PME) method^78^

### Umbrella sampling

The umbrella sampling method was used to calculate the potential of mean force (PMF) of selected molecules along the reaction coordinate. The reaction coordinate was defined as the distance between the center of mass (COM) of the membrane center and the COM of the antimicrobial agent, projected along the bilayer normal (the *z* direction). First, a steered molecular dynamics simulation was performed to collect the initial configurations for the umbrella sampling method. Here, the antimicrobial agent was pulled toward the bilayer center from bulk water. To provide adequate sampling, a minimum of 40.000 samples were collected for each step, with a 0.1nm width spacing taken between subsequent windows. In some cases, additional simulations were carried out to improve the sampling. Further, a brief (10ns) NVT and NPT equilibration was carried out for each window. Subsequently, proper umbrella sampling simulations were performed with 1000 kJ mol$^{-1}$ nm$^{-2}$ force constants using the harmonic potential, the restraining agent along the reaction coordinate. Each window was simulated for 50ns. Subsequently, the weighted histogram analysis method (WHAM), built into the GROMACS software, was employed to calculate the free energy profile^79^.

### Supplementary Information

Supplementary Information 1.

Supplementary Information 2.

## Data Availability

Diptool is freely available in a GitHub repository (https://github.com/mrzyckiz/Diptool). The database of all antimicrobial agents with the corresponding PDBs and force-field files is available at https://doi.org/10.3390/ijms222010939.
