# Supplementary material for: Molecular guidelines for promising antimicrobial agents
Source: Sci Rep. 2024 Feb 26;14:4641. doi: 10.1038/s41598-024-55418-6 (PMC11322663; doi:10.1038/s41598-024-55418-6)
Supplement: Supplementary file 1 — Supplementary Information 1. [file 41598_2024_55418_MOESM1_ESM.pdf]

Supporting Information: Molecular Guidelines for Promising Antimicrobial Agents

Mateusz Rzycki[1,*], **Marta Gadysiewicz-Kudrawiec**[2], **and Sebastian Kraszewski**[1]

[1]Department of Biomedical Engineering, Wroclaw University of Science and Technology, Wroclaw, 50-370, Poland
[2]Department of Experimental Physics, Wroclaw University of Science and Technology, Wroclaw, 50-370, Poland
*mateusz.rzycki@pwr.edu.pl

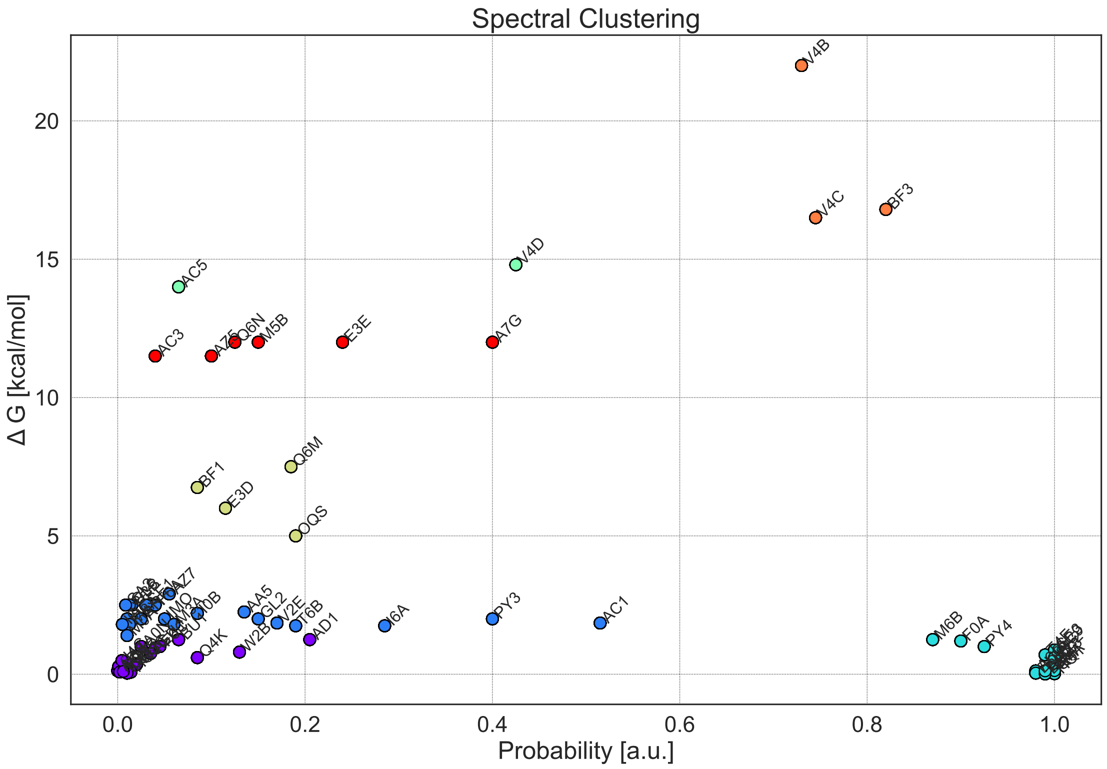

Figure S1. Spectral clustering of all antimicrobial candidates collected in the study based on their Diptool dG and probability of entry. The clustering process identified seven distinct groups, sequentially marked with individual colors.

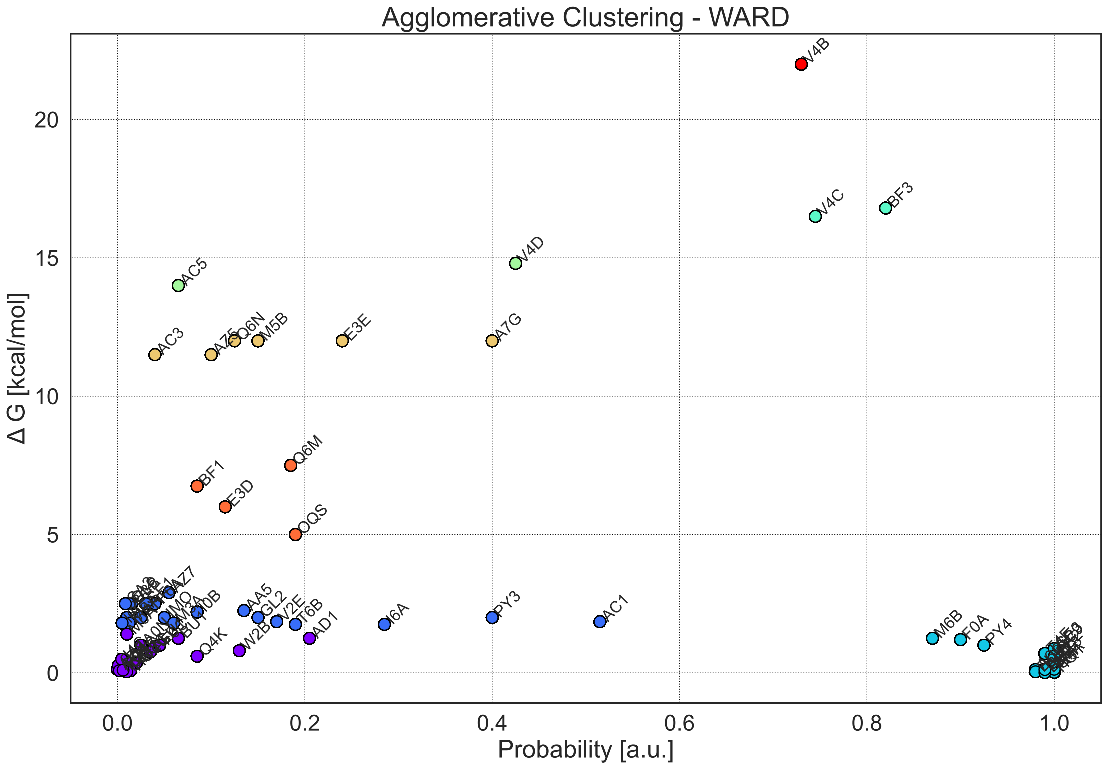

Figure S2. Agglomerative clustering of all antimicrobial candidates collected in the study based on their Diptool dG and probability of entry. The clustering process identified eight distinct groups, sequentially marked with individual colors.

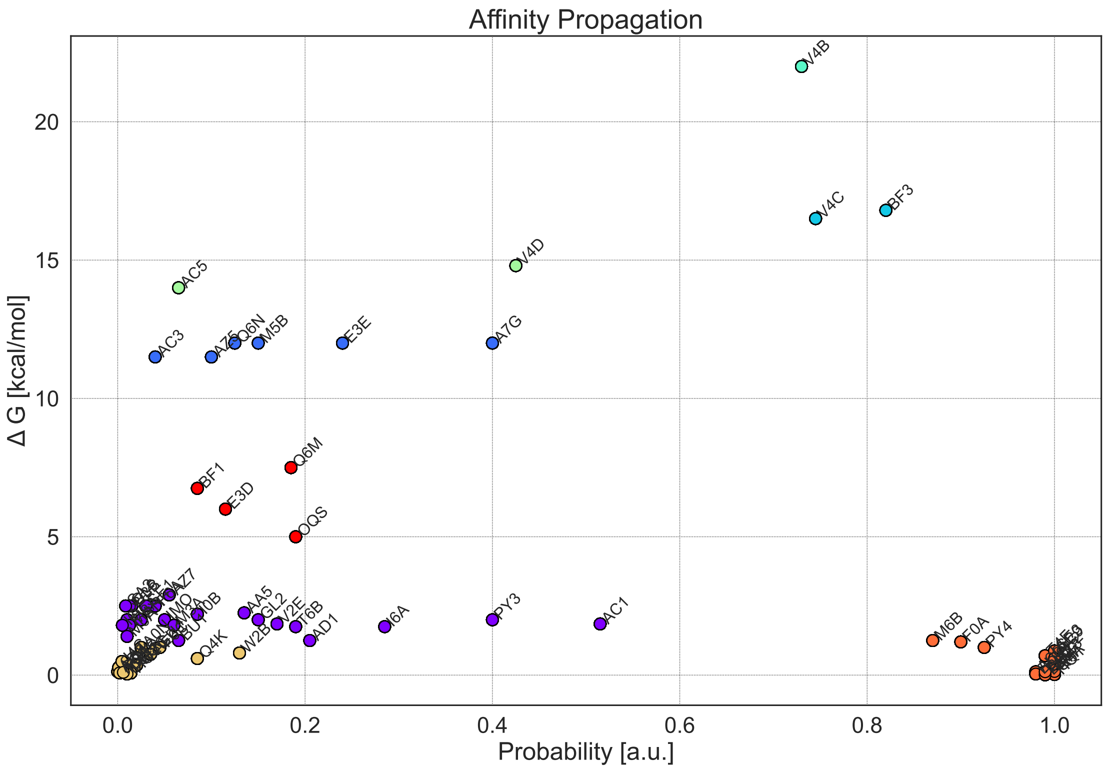

Figure S3. Affinity clustering of all antimicrobial candidates collected in the study based on their Diptool dG and probability of entry. The clustering process identified eight distinct groups, sequentially marked with individual colors.

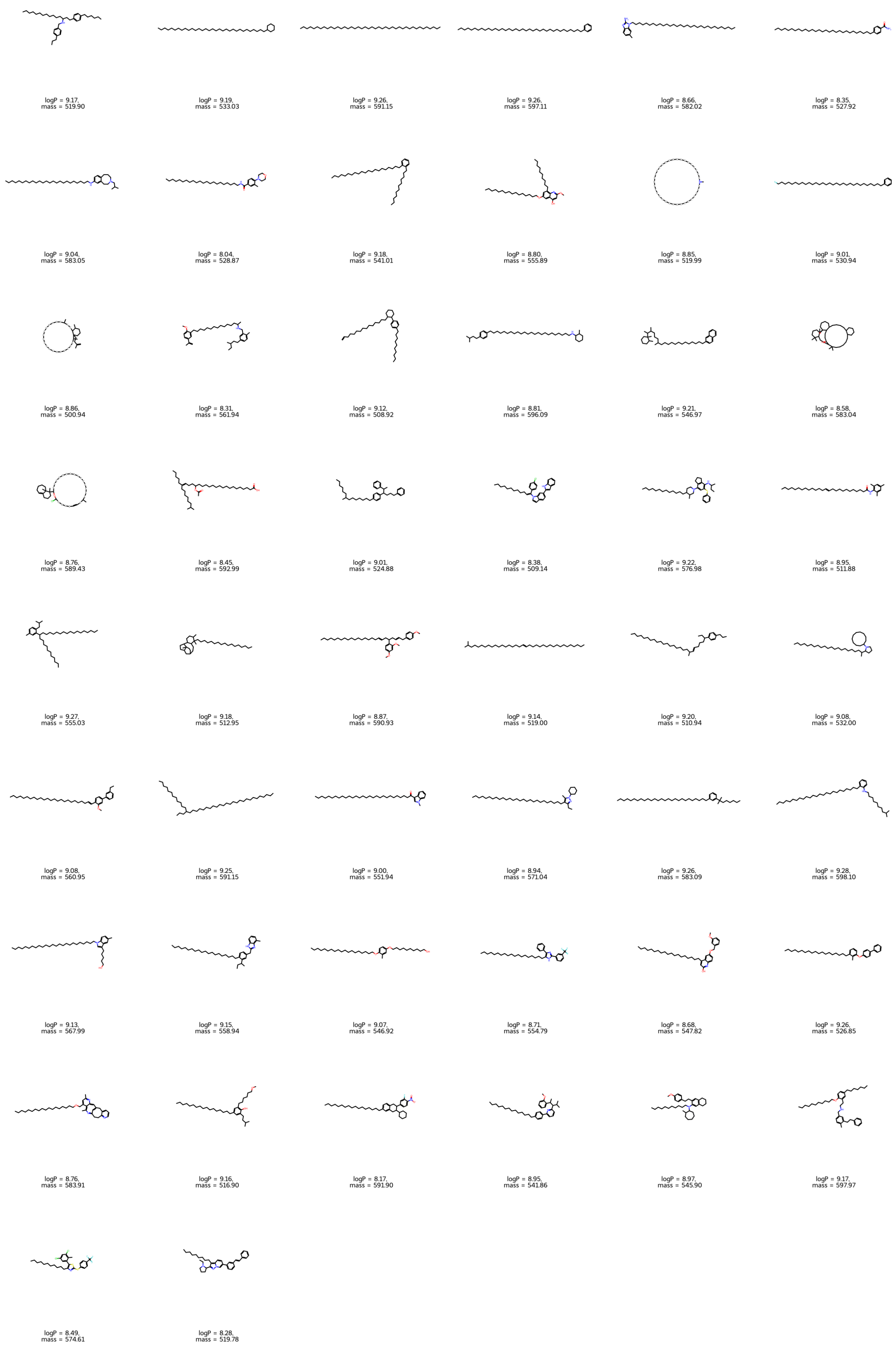

Figure S4. A set of fully AI-formed molecules using the ReLeaSE method, based on the derived logP and mass guidelines.
